# Brief Remote Intervention to Manage Food Cravings and Emotions During the COVID-19 Pandemic: A Pilot Study

**DOI:** 10.3389/fpsyg.2022.903096

**Published:** 2022-06-30

**Authors:** Tracey J. Devonport, Chao-Hwa Chen-Wilson, Wendy Nicholls, Claudio Robazza, Jonathan Y. Cagas, Javier Fernández-Montalvo, Youngjun Choi, Montse C. Ruiz

**Affiliations:** ^1^Sport and Physical Activity Research Centre, University of Wolverhampton, Wolverhampton, United Kingdom; ^2^Faculty of Health, Education & Society, University of Northampton, Northampton, United Kingdom; ^3^Faculty of Education, Health and Wellbeing, University of Wolverhampton, Wolverhampton, United Kingdom; ^4^Behavioral Imaging and Neural Dynamics Center, G. d'Annunzio University of Chieti–Pescara, Chieti, Italy; ^5^Department of Sports Science, University of the Philippines Diliman, Quezon, Philippines; ^6^Faculty of Health Sciences, Universidad Pública de Navarra, Pamplona, Spain; ^7^Department of Physical Education, Jeju National University, Jeju, South Korea; ^8^Faculty of Sport and Health Sciences, University of Jyväskylä, Jyväskylä, Finland

**Keywords:** lockdown, confinement, mindful eating, diary, emotion

## Abstract

As a result of the COVID-19 pandemic people have endured potentially stressful challenges which have influenced behaviors such as eating. This pilot study examined the effectiveness of two brief interventions aimed to help individuals deal with food cravings and associated emotional experiences. Participants were 165 individuals residing in United Kingdom, Finland, Philippines, Spain, Italy, Brazil, North America, South Korea, and China. The study was implemented remotely, thus without any contact with researchers, and involved two groups. Group one participants were requested to use daily diaries for seven consecutive days to assess the frequency of experience of their food cravings, frequency of giving in to cravings, and difficulty resisting cravings, as well as emotional states associated with their cravings. In addition to completing daily food diaries, participants in group two were asked to engage in mindful eating practice and forming implementation intentions. Participants assessed their perceived changes in eating, wellbeing, and health at the beginning and end of the intervention. Repeated measures MANOVAs indicated that participants experienced significantly less food cravings (i.e., craving experience, acting on cravings, difficulty resisting), as well as lower intensities of unpleasant states associated with cravings across time (T1 vs. T7). In contrast to our hypothesis, the main effects of the group (food craving diary vs. food craving diary and mindful eating practice) were not significant. Participants reported less eating and enhanced wellbeing at the end of the study (T7 vs. T1). Our findings can be used to inform future remote interventions to manage food cravings and associated emotions and highlight the need for alternative solutions to increase participant engagement.

## Introduction

On March 11th 2020, the World Health Organization declared COVID-19 a pandemic, and by September 22nd 2021 there have been nearly 230 million cases and over 4.7 million deaths worldwide (World Health Organisation, 2021). Being highly contagious, a common containment measure adopted by many nations during the pandemic was a mandated “lockdown”. This resulted in the closure of schools, businesses and places of congregation, and travel restrictions. During this time, people were allowed to leave their homes only to purchase essential items (e.g., food, medicines), seek essential treatment, go to work (only for jobs considered essential), or to assist and care for dependents. Adjusting to life during the COVID-19 pandemic presented considerable challenges such as home-schooling, learning new ways of working, reduced opportunity to pursue hobbies dependent on congregating, and lost time spent with friends and family.

An increase in stress, depression, anxiety, and other unpleasant emotions was reported as a consequence of the COVID-19 pandemic (Salari et al., 2020). Also evidenced has been the experience of anxiety due to poor eating habits with individuals reporting using food to feel better (Di Renzo et al., 2020). Notably, increased calorie consumption derived largely from foods high in fats and sugars, thus leading to weight gain (Shen et al., 2020). Increasing calorie intake via unhealthy food choices when experiencing unpleasant emotions in the absence of internal hunger cues is termed emotional eating (van Strien et al., 2007). However, whilst emotional eating was originally defined as eating in response to unpleasant emotions, a number of studies show that pleasant emotions can also elicit increased food intake (Nicholls et al., 2016; Devonport et al., 2019). Therefore, in the present study we considered both unpleasant and pleasant emotions.

Exposure to stress during the pandemic, and the subsequent potential for unpleasant emotions and associated weight gain, increases risk of adverse outcomes from COVID-19. Results from a survey administered to 1,140 individuals residing in different countries showed an increase in reported eating and weight, especially for those reporting highest decrease in physical activity as a consequence of the COVID-19 pandemic (Ruiz et al., 2021). Targeting eating behavior through provision of remote interventions that could be easily administered and would not require the presence of a practitioner is, therefore, a critical strategy to ameliorate the impact of COVID-19 and is the focus of the proposed research.

Traditional calorie-controlled diets can lead to individuals feeling deprived of food, which can result in food cravings and decreased sensitivity to sensations of hunger and fullness (Meule, 2020). Interventions that take account of the psychological processes underpinning eating behavior may have greater long-term success than nutritional only interventions (Braden et al., 2016). For example, a food craving is an intense desire for specific foods that are difficult to resist, and a leading cause of dieting failure (Meule, 2020). Cravings present a non-hunger cue relevant to the COVID-19 pandemic situation. Eating behavior interventions typically address cravings through the avoidance or removal of cues or situations which are known triggers (Nannt et al., 2019). However, during lockdown, people have been purchasing high quantities of long-life food high in sugar, trans fat, and salt content, which contribute to obesity (Rundle et al., 2020). Such changes in food purchasing make it difficult to address food cravings through cue avoidance, as individuals are living with larger quantities of unhealthy food within their immediate environment.

In selecting interventions for use in the present study, there was a need to be sensitive to considerations of participant need and participant burden. The term “zoom fatigue”, which gained momentum during COVID-19, reflects increased personal, professional, and psychological demands resulting from higher use of technology for work and social purposes (Brown et al., 2021). The ongoing pandemic necessitated the use of online means for facilitating remote interventions. Cognisant of a need to account for the possibilities of zoom fatigue and increased demands resulting from home working, schooling, carer responsibilities, etc., we sought to deliver brief interventions previously established as effective in regulating unpleasant emotions and resisting food cravings when delivered remotely. Specifically, in our remote intervention we implemented food craving diaries and mindful eating techniques and recorded the type of food individuals gave into after cravings.

Evidence suggests that maintaining a daily eating diary is associated with significantly more weight loss than an inconsistent recording (Berkowitz et al., 2003). This is because a daily diary increases awareness of eating habits, and when the focus is on emotional antecedents of food cravings, self-monitoring can provide a more complete understanding of eating behaviors. Not only can this help in targeting eating interventions appropriately, but the diary in itself also presents an intervention due to its role in increasing awareness (Jimoh et al., 2018).

Mindful eating is based on the construct of mindfulness, which refers to paying attention on purpose, non-judgmentally, and in the present moment (Kabat-Zinn, 1990). While some scholars consider mindfulness a skill that can be practiced (e.g., Bishop et al., 2004), others consider it a dispositional trait present in every person (Brown and Ryan, 2003). Mindful eating is viewed as a process that involves three aspects: (1) bringing attention to present moment experience, that is, the process of eating, taste, smells, thoughts, and feelings that arise during a meal, as well as internal cues of hunger and fullness; (2) considering one's thoughts and emotions as separate from oneself, also called decentering; and (3), acceptance of one's experiences, also called non-reactivity (Tapper, 2022).

Most common mindful eating practices include present moment awareness of the sensory properties of food, internal bodily sensations or cues that elicit eating or the urge to eat, and decentering from or acceptance of cravings or food-related thoughts (Tapper, 2022). Because awareness of one's emotions is necessary for successful emotion regulation, mindful eating would appear suitable in facilitating the management of emotionally elicited eating. Support for this contention comes from research demonstrating that mindfulness treatment is linked to anatomical changes in areas associated with cortical regulation and emotion regulation (Hölzel et al., 2011). Research also suggests that mindful eating reduces impulsive eating in response to emotional stressors and re-engages intuitive processes of eating regulation (Kristeller and Wolever, 2010). Focusing on sensory experiences (e.g., taste and texture), increases awareness of satiety resulting in reduced consumption of sweet foods (Mason et al., 2016) and lower calorie intake (Arch et al., 2016).

Forming implementation intentions is a self-regulation strategy that involves a volitional process and is thus concerned with filling the gap between intention and behavior by planning the action to achieve a certain goal (Gollwitzer, 1996; Bieleke et al., 2021). Such strategy, which takes the if-then format - “*If* (situation), *then* I will do (behavior)”, helps individuals specify when, where, and how to perform goal-directed responses. Research has shown that forming implementation intentions is an effective strategy in the reduction of fat consumption (Vilà et al., 2017) and in modifying emotional outcomes (Webb et al., 2012).

Widespread use of the internet, smartphones, and mobile technology (Roberts et al., 2017) have enabled the rapid growth of brief online health and mental health programmes, including mindful eating training (Mason et al., 2018) and food craving diaries (Schumacher et al., 2018). Implementing interventions online not only circumnavigates the challenges of intervention delivery whilst social distancing measures are in place, but also presents scope for reaching a wider audience (Moller et al., 2017). Previous research shows that remote interventions can reduce unhelpful eating behaviors (Mason et al., 2018; Schumacher et al., 2018). A consideration in delivering remote intervention studies is that participants are truly volunteers. There is no associative incentive for participation, or power differentials influencing participation that are omnipresent in research undertaken by academics using student populations. The implication being that they have given thought to their circumstances, their emotions and eating, and made a commitment to attempt change. Thus, this presents an opportunity to evaluate the acceptability of interventions provided and establish impact.

### Study Purpose

The purpose of this pilot study was to explore the effectiveness of a seven-day remote intervention to manage food cravings during the COVID-19 pandemic. The intervention included two conditions, (a) completion of a food craving diary and (b) completion of diary and mindful eating practice. Food craving diaries and emotional states assessments were provided with the intention of helping individuals identify emotional antecedents of food cravings. Mindful eating guidelines were provided to better manage food cravings when they inevitably did occur. Both conditions sought to challenge eating in response to non-hunger cues, specifically emotions, known to be associated with snacking on highly fatty and sugary food (Summerbell et al., 1995). Differences in food cravings, emotional states associated with cravings, changes in eating, perceived wellbeing, and physical and mental health were examined. Based on previous experimental evidence on the effectiveness of mindful eating on energy intake (Tapper, 2022) and implementation intentions (Bieleke et al., 2021), it was hypothesized that a mindful eating practice combined with the completion of a food-craving diary would be more effective in helping individuals manage their food cravings and related emotional experiences than the completion of the food-craving diary alone, which would result in decreased eating and increased perceptions of wellbeing, physical and mental health.

## Materials and Methods

### Participants and Procedures

An a priori sample size calculation for repeated measures MANOVA design with an anticipated medium effect size of 0.30, power level of 0.80, and *P* < 0.05 for two groups and seven measurement times, suggested a minimum sample size of 158 participants (G^*^Power 3.1.9.6 software; Faul et al., 2007). The recommended sample for the between-subjects main effect (correlation among repeated measures = 0.5) was 52 participants, and for the within-subjects main effect 20 participants. The only criterion for participation in the study was being over 18 years of age.

**Figure 1:**
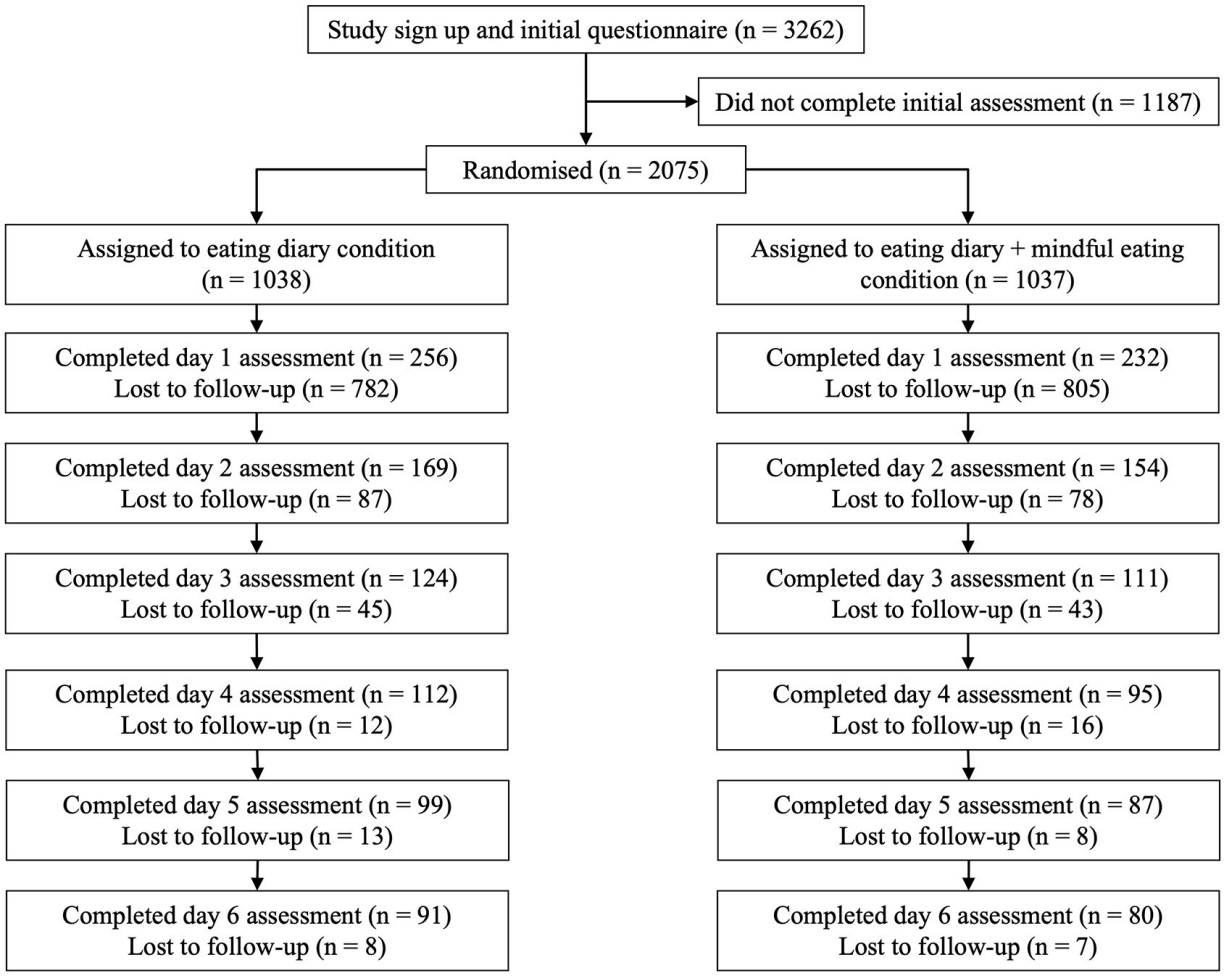
Flow diagram representing participants' attrition from the survey and follow-up assessments.

Up to 3,262 participants started the initial assessments. Of the 2,075 who completed the initial assessments, 171 completed the study (see Figure 1). Data screening suggested the removal of six cases identified as outliers using the Mahalanobis' distance criterion. The final sample consisted of 165 participants including women (*n* = 132), men (*n* = 32), and one person who identified as other gender. Participants were aged 18 to 72 years (*M* = 33.64, *SD* = 12.42) and resided in different countries (i.e., UK 24%, Finland 23%, Philippines 22%, Spain 12%, Italy 8%, Brazil or Portugal 4%, North America 4%, South Korea 2%, and China 1%).

The study was conducted between May and November 2020, completely online; thus, there was no contact between participants and researchers. The study was implemented using Qualtrics. Links to the study were distributed via social media (e.g., Twitter, Facebook, LinkedIn), and other public channels (e.g., Universities). Participants were presented details about the purpose and protocol, and assurances of anonymity and confidentiality, after which they provided informed consent, which was granted electronically. Participation was voluntary and no compensation for taking part in the study was given. Participants could exercise their right to withdraw from the study by exiting the browsing window. Ethics approval was granted by the University of Wolverhampton (01/20/AF1/UOW).

Separate Qualtrics projects were developed to include information in the respective languages of the participants. Instructions, measures, and guidelines were translated from English into other languages (i.e., Finnish, Filipino, Spanish, Italian, Portuguese, Korean, and Chinese) using back translation procedures (Brislin, 1986). This comprised (a) translation into the respective languages by bilingual individuals with efforts made to include most common or used language, (b) back-translation into English, (c) comparison of translated and original versions by experts with the focus on maintaining the meaning of the original texts.

This randomized pilot study was a pre-test post-test research design, including two conditions, and seven consecutive assessments (T1 through T7) divided in three phases: (1) initial assessment, (2) follow up assessments, and (3) evaluation.

### Measures

#### Phase 1: Initial Assessment

Participants provided demographic information such as age, gender, country of residence, and ethnicity. In addition, they completed the following measures at the beginning and at the end of the study.

##### Perceived Changes in Eating and Wellbeing

Two single items were used to assess participants' changes in their eating and wellbeing respectively, as consequence of the COVID-19 pandemic. Participants were asked to rate changes in eating by answering to the stem question “I feel that I currently eat…” on a 11-point scale ranging from −5 (*less*) to +5 (*more*), with 0 indicating *no change*. Participants assessed their perceptions of wellbeing by responding to the question “I feel that my wellbeing is…” on an 11-point scale ranging from −5 (*significantly decreased*) to +5 (*significantly increased*), with 0 indicating *not changed*. These two items were developed for another study that explored changes in working situation, health routines, and wellbeing in a larger sample (Ruiz et al., 2021). Single items were used as a viable alternative to reduce burden and increase willingness to complete the measures (Allen et al., 2022).

##### Physical and Mental Health

Two items from the SF-8 Health Survey (Ware et al., 2001) measured participants' physical and mental health. Specifically, physical health was measured by the following item “Overall, how would you rate your health during the past week?” which was rated on a six-point scale ranging from 1 (*very poor*) to 6 (*excellent*). As an indicator of mental health, using the same timeframe of “during the past week”, a second item asked participants to assess “How much did personal or emotional problems keep you from doing your usual work, school or other daily activities?” The following anchors were used: 1 (*not at all*), 2 (*very little*), 3 (*somewhat*), 4 (*quite a lot*), and 5 (*could not do daily activities*).

##### Food Craving Diary

A food craving diary was developed including three questions. The first question assessed the frequency of food cravings experienced (i.e., “How often have you experienced a food craving today?”) and was rated using the following anchors: 0 (*never*), 1 (*rarely*), 2 (*sometimes*), 3 (*often*), 4 (*always*). The second question measured the frequency of participants giving in to cravings (i.e., “How often did you give in to cravings and eat the food today?”) and was rated using the following anchors: 0 (*never*), 1 (*rarely*), 2 (*sometimes*), 3 (*often*), 4 (*almost every time*). The third question assessed participants' difficulty resisting the craving (i.e., “How difficult was it to resist temptation?”) which was rated on the following anchors: 0 (*easy*), 1 (*a bit difficult*), 2 (*difficult*), 3 (*very difficult*), 4 (*so difficult that I gave in*). In addition, participants were asked to indicate whether the most common type of food ingested following cravings were sweet foods (e.g., cake, biscuits, confectionary, sweets) or savory foods (e.g., crisps, popcorn, pretzels, meat snacks). In the current study, acceptable reliability was found for the three first questions for participants who completed the first assessment (*n* = 2075) with a Cronbach alpha value (α) of 0.789, and McDonalds' omega (ω) value of 0.792.

##### Emotional States Associated With Food Cravings

Participants were requested to rate the intensity of nine emotional states (i.e., energetic, angry, anxious, happy, relaxed, miserable, tired, bored, and frustrated) to indicate their experiences at the time of the strongest food craving. These emotions represent the hedonic valence (pleasure-displeasure) and activation (high-low) described in Russell (1980) circumplex model of affective experiences. The intensity of these emotional states was rated on a 6-point Likert scale: 0 (*none at all*), 1 (*a little*), 2 (*moderately*), 3 (*quite a bit*), 4 (*a lot*), 5 (*a great deal*). If the emotion they experienced at the time of the strongest craving was not included in the list, they could indicate their own.

#### Phase 2: Follow up Assessments

After participants indicated their gender (male, female, other), they were randomly assigned to either food craving diary (FCD) group (*n* = 88) or food craving diary and mindful eating (FCDM) group (*n* = 77) within their chosen gender category (see Figure 1). This was done for each participating country.

FCD group participants completed the food craving diary and assessed their emotional states associated with food cravings for six consecutive days. In addition to doing this, FCDM group participants were provided guidelines (see Section Mindful Guidelines Before Eating and and Mindful Guidelines During Eating) to follow before and during eating for mindful eating practice. Participants were requested to provide their email address to receive daily project reminders and a link to follow-up surveys for the duration of the study. To ensure anonymity, automatic reminders were created within the Qualtrics platform and were sent out at the end of the following day. Follow-up surveys were sent at around 8 pm and included the food diary for FCD group, and food diary and mindful eating guidelines for FCDM group. Mindful eating guidelines, which participants were encouraged to follow every day, were available to download each day.

##### Mindful Guidelines Before Eating

One aspect of mindful eating is to recognize and accept the ebb and flow of different emotions that might lead to emotional eating. Having completed the food craving diary, think about the connections between your emotions and a desire to eat. For example, did you experience food cravings when feeling lonely, sad, angry, disappointed, excited, anxious, bored, or guilty/ashamed? It might help you to understand emotional triggers for eating if you think about how you want to feel during/after eating. For example: If you want to feel more energized, this might indicate the trigger is tiredness; if you want to feel soothed/calm/relaxed, this might indicate the trigger is some kind of anxiety or anger; if you want to feel distracted, this might indicate the trigger is frustration, disappointment, loneliness, anger, or anxiety; if you want to feel entertained, this might indicate the trigger is boredom or loneliness. Sometimes understanding why you have experienced a food craving is enough to resist the craving. Sometimes understanding why you have experienced a food craving gives you the information you need to pick a different coping response that's a better match to the problem you're trying to solve. Think of different ways that you can manage emotions other than eating. For example, you are bored so call a friend. For common triggers for emotional eating, develop a plan that says… when I feel X, doing A, B, C, or D is likely to help. For example, if I feel anxious, then I can listen to music, watch a favorite film, speak with a close friend…

##### Mindful Guidelines During Eating

The development of mindful eating involves bringing full attention to the process of eating, to taste, smells, thoughts, and feelings that arise during a meal, as well as internal cues of hunger and fullness. To practice mindful eating: (a) focus on noticing food and your body's response to eating; (b) slow down when you are eating; and (c) take time to savor and enjoy your food and notice textures and flavors.

#### Phase 3: Evaluation

Participants were asked to assess their perceived changes in eating and wellbeing and their physical and mental health. In addition, they assessed perceived easiness and effectiveness.

##### Perceived Easiness

Participants were asked to rate the ease of completing the food craving diary. In addition, FCDM participants were also asked to rate the ease of undertaking mindful eating practice. Easiness was rated on a 7-point scale ranging from −3 (*I found it hard to do*) to +3 (*I found it very easy to do*), or 0 (*I did not do it*).

##### Perceived Effectiveness

Participants were requested to assess the effectiveness of the food craving diary, and for FCDM participants also the mindful eating practice in helping them to manage food cravings. Effectiveness was rated on a 7-point scale ranging from −3 (*not at all effective*) to +3 (*very effective*), or 0 (*hard to say*).

### Statistical Analysis

Prior to conducting the main analysis, potential differences in mean scores in the study variables at Time 1 (T1) across groups (FCD vs. FCDM) were examined through multivariate analysis of variance (MANOVA). The main analysis comprised four repeated measures MANOVAs to examine: (1) differences in food cravings, (2) emotional states associated with cravings, (3) perceived changes in eating and wellbeing, and physical and mental health, and (4) easiness to follow intervention and intervention effectiveness. The first repeated measures MANOVA examined differences in food cravings data with Time (T1, T2, T3, T4, T5, T6, T7) as a within-subjects factor and Group (FCD, FCDM) as a between-subjects factor, and experience of food cravings, frequency in eating after cravings, and difficulty resisting cravings as outcome variables. A second repeated measures MANOVA was conducted to examine differences in the emotional states (i.e., energetic, angry, anxious, happy, relaxed, miserable, tired, bored, and frustrated) experienced at the time of strongest cravings. Emotional states were included in the analysis separately. A third repeated measures MANOVA was performed to examine differences in perceived changes in eating and wellbeing, and physical and mental health across intervention (T1 vs. T7) and groups. A fourth repeated measures MANOVA was conducted to examine the differences in perceived easiness as well as effectiveness of the food diary across groups.

## Results

### Preliminary Analysis

Regarding potential differences in mean scores in the study variables at Time 1, MANOVA demonstrated equivalence between groups indicating no significant differences across groups for food cravings experienced, frequency of giving in to cravings, difficulty resisting cravings, or any of the emotional states experienced at T1 Wilk's λ = 0.926, *F*_(12, 152)_ = 1.014, *P* = 0.439, $\eta_{\text{p}}^{2}$ = 0.074.

Adequate reliability for food cravings experience, frequency of giving in to cravings, and difficulty resisting cravings from T1 through T7 was found with α > 0.799 and ω > 0.813. The most common types of food consumed after giving into cravings were sweet foods (e.g., cake, biscuits, confectionary, sweets) prior to the intervention (71% of participants) and at the end of the intervention (64%).

There were significant differences in the completion rate for gender (women, men) by group (food craving diary, food craving diary + mindful eating) across the seven days, $\chi_{\left(1,2048\right)}^{2}$ = 12.55, *P* < 0.001, with men withdrawing from the study at higher rates than women for both groups (see also Figure 1).

### Food Cravings and Emotional States

Descriptive statistics for food cravings and emotional states by experimental groups (FCD vs. FCDM) are presented in Table 1 (Pearson product-moment correlation coefficients at T1 are presented in Supplementary Table S1).

**Table 1:** Descriptive statistics of food cravings and emotions for participants in FCD group (*n* = 88) and FCDM group (*n* = 77).

	**T1**	**T2**	**T3**	**T4**	**T5**	**T6**	**T7**
	***M (SD)***	***M (SD)***	***M (SD)***	***M (SD)***	***M (SD)***	***M (SD)***	***M (SD)***
**FCD group**						
Food cravings						
Craving frequency	3.2 (0.9)	2.5 (0.9)	2.5 (1.0)	2.4 (1.0)	2.5 (1.1)	2.4 (1.0)	2.5 (1.0)
Give into craving	3.1 (1.1)	2.4 (1.1)	2.6 (1.2)	2.4 (1.2)	2.3 (1.4)	2.4 (1.3)	2.3 (1.3)
Difficulty resisting	2.7 (1.2)	2.1 (1.2)	2.3 (1.4)	2.2 (1.3)	2.2 (1.3)	2.3 (1.3)	2.2 (1.3)
Emotional states						
Energetic	1.1 (1.3)	1.2 (1.3)	1.0 (1.2)	1.1 (1.4)	1.0 (1.3)	1.0 (1.3)	1.2 (1.3)
Angry	0.7 (1.0)	0.4 (0.8)	0.5 (1.0)	0.4 (1.0)	0.3 (0.7)	0.4 (0.8)	0.5 (0.9)
Anxious	1.3 (1.6)	1.1 (1.4)	0.8 (1.4)	0.8 (1.3)	0.9 (1.5)	0.9 (1.4)	0.9 (1.4)
Happy	1.3 (1.4)	1.4 (1.4)	1.3 (1.2)	1.5 (1.5)	1.3 (1.3)	1.2 (1.4)	1.2 (1.3)
Relaxed	1.2 (1.3)	1.4 (1.3)	1.3 (1.3)	1.4 (1.3)	1.1 (1.2)	1.2 (1.3)	1.1 (1.3)
Miserable	1.1 (1.5)	0.7 (1.3)	0.7 (1.2)	0.6 (1.2)	0.8 (1.4)	0.7 (1.3)	0.7 (1.2)
Tired	1.7 (1.7)	1.5 (1.6)	1.6 (1.6)	1.5 (1.6)	1.6 (1.7)	1.5 (1.6)	1.5 (1.6)
Bored	1.9 (1.7)	1.5 (1.6)	1.3 (1.6)	1.2 (1.6)	1.0 (1.4)	1.0 (1.4)	0.9 (1.3)
Frustrated	1.3 (1.6)	0.9 (1.4)	1.0 (1.5)	0.7 (1.3)	0.8 (1.3)	0.7 (1.3)	0.9 (1.4)
**FCDM group**						
Food cravings						
Craving frequency	3.2 (1.0)	2.8 (1.0)	2.6 (1.1)	2.3 (0.9)	2.2 (0.9)	2.4 (1.0)	2.3 (1.0)
Give into craving	3.0 (1.1)	2.7 (1.2)	2.3 (1.3)	2.2 (1.2)	2.2 (1.2)	2.3 (1.2)	2.2 (1.2)
Difficulty resisting	2.6 (1.1)	2.4 (1.2)	2.1 (1.3)	2.0 (1.2)	2.1 (1.2)	2.2 (1.2)	2.1 (1.3)
Emotional states						
Energetic	1.1 (1.3)	0.9 (1.1)	0.9 (1.2)	1.2 (1.2)	0.9 (1.1)	1.0 (1.2)	0.9 (1.1)
Relaxed	1.6 (1.4)	1.2 (1.3)	1.4 (1.6)	1.2 (1.3)	1.2 (1.2)	1.3 (1.3)	1.1 (1.2)
Happy	1.2 (1.3)	1.1 (1.4)	1.4 (1.3)	1.2 (1.4)	1.1 (1.2)	1.3 (1.3)	1.1 (1.4)
Anxious	1.5 (1.7)	1.3 (1.5)	0.9 (1.2)	0.7 (1.1)	0.8 (1.2)	0.7 (1.1)	0.9 (1.3)
Angry	0.7 (1.1)	0.7 (1.1)	0.5 (1.0)	0.4 (0.7)	0.4 (1.0)	0.4 (0.8)	0.4 (0.8)
Frustrated	1.4 (1.6)	1.3 (1.6)	1.0 (1.5)	0.7 (1.2)	0.6 (1.1)	0.6 (1.0)	0.7 (1.2)
Miserable	1.2 (1.3)	1.0 (1.4)	0.7 (1.2)	0.5 (1.0)	0.6 (1.2)	0.5 (1.0)	0.6 (1.1)
Tired	2.0 (1.6)	1.9 (1.4)	1.4 (1.6)	1.4 (1.5)	1.3 (1.4)	1.3 (1.4)	1.2 (1.4)
Bored	2.2 (1.6)	1.3 (1.6)	1.3 (1.6)	1.1 (1.4)	1.1 (1.4)	1.0 (1.4)	0.9 (1.3)

**Figure 2:**
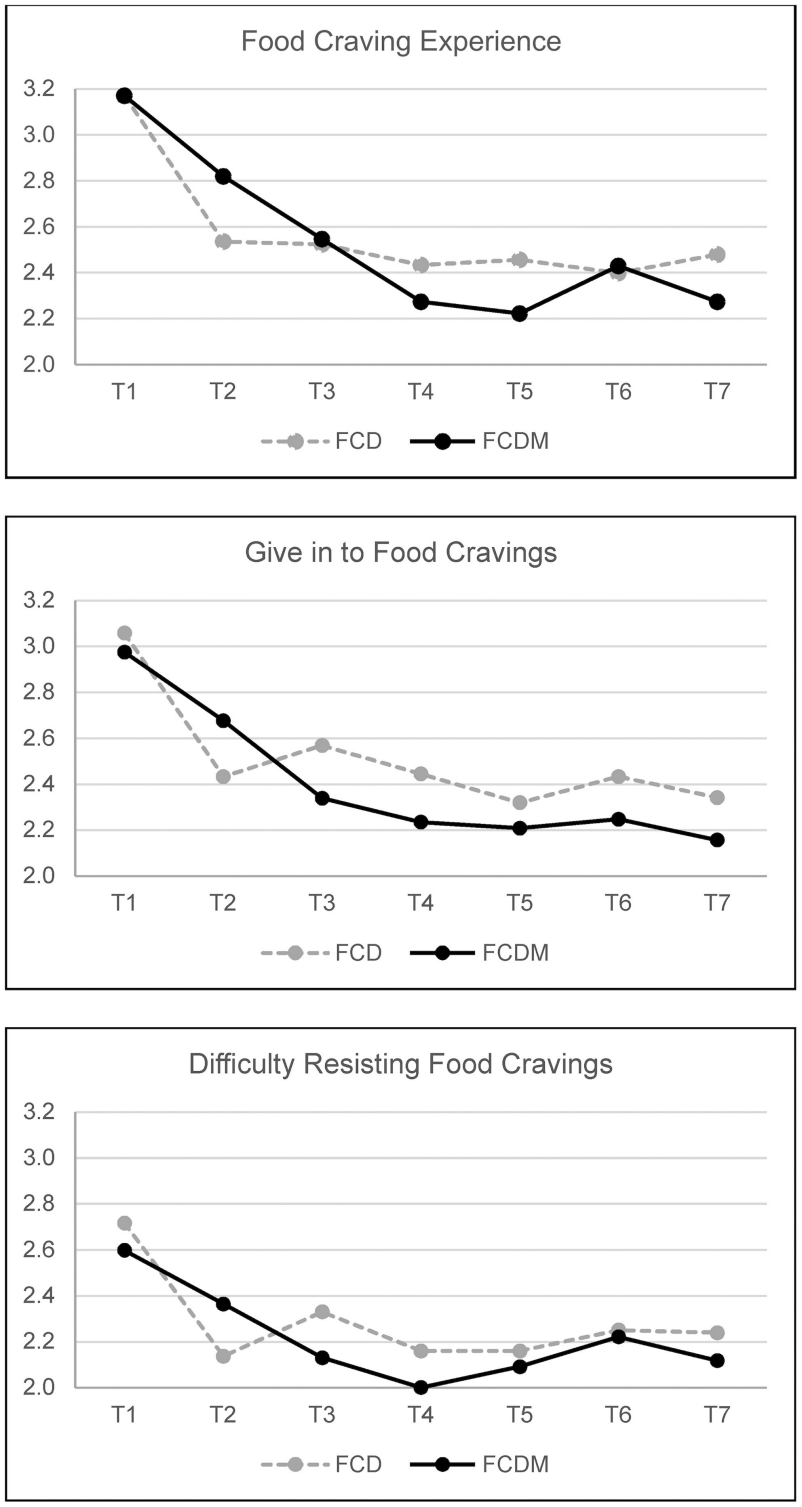
Food cravings across a 7-day intervention (FCD, *n* = 88; FCDM, *n* = 77).

Regarding differences in food cravings, as expected, repeated measures MANOVA yielded significant main effects of Time, Wilk's λ = 0.465, *F*_(18, 146)_ = 9.334, *P* < 0.001, $\eta_{\text{p}}^{2}$ = 0.535 for food cravings (i.e., cravings experienced, giving in to cravings, and difficulty resisting the cravings). Contrary to our hypothesis, the effect of Group was not significant, Wilk's λ = 0.993, *F*_(3, 161)_ = 0.376, *P* = 0.770, $\eta_{\text{p}}^{2}$ = 0.007. The effect of Time by Group interaction was also not significant. Ratings of the three craving aspects for each group across the seven days are depicted in Figure 2. *Post hoc* analysis on the main effects of Time revealed significantly lower values at the end of the intervention in the frequency of cravings experienced, *F*_(1, 163)_ = 46.268, *P* < 0.001, $\eta_{\text{p}}^{2}$ = 0.221, in giving in to cravings, *F*_(1, 163)_ = 38.975, *P* < 0.001, $\eta_{\text{p}}^{2}$ = 0.193, and in the difficulty in resisting the cravings, *F*_(1, 163)_ = 10.174, *P* = 0.002, $\eta_{\text{p}}^{2}$ = 0.059 (see Supplementary Table S2 for pairwise comparisons).

**Figure 3:**
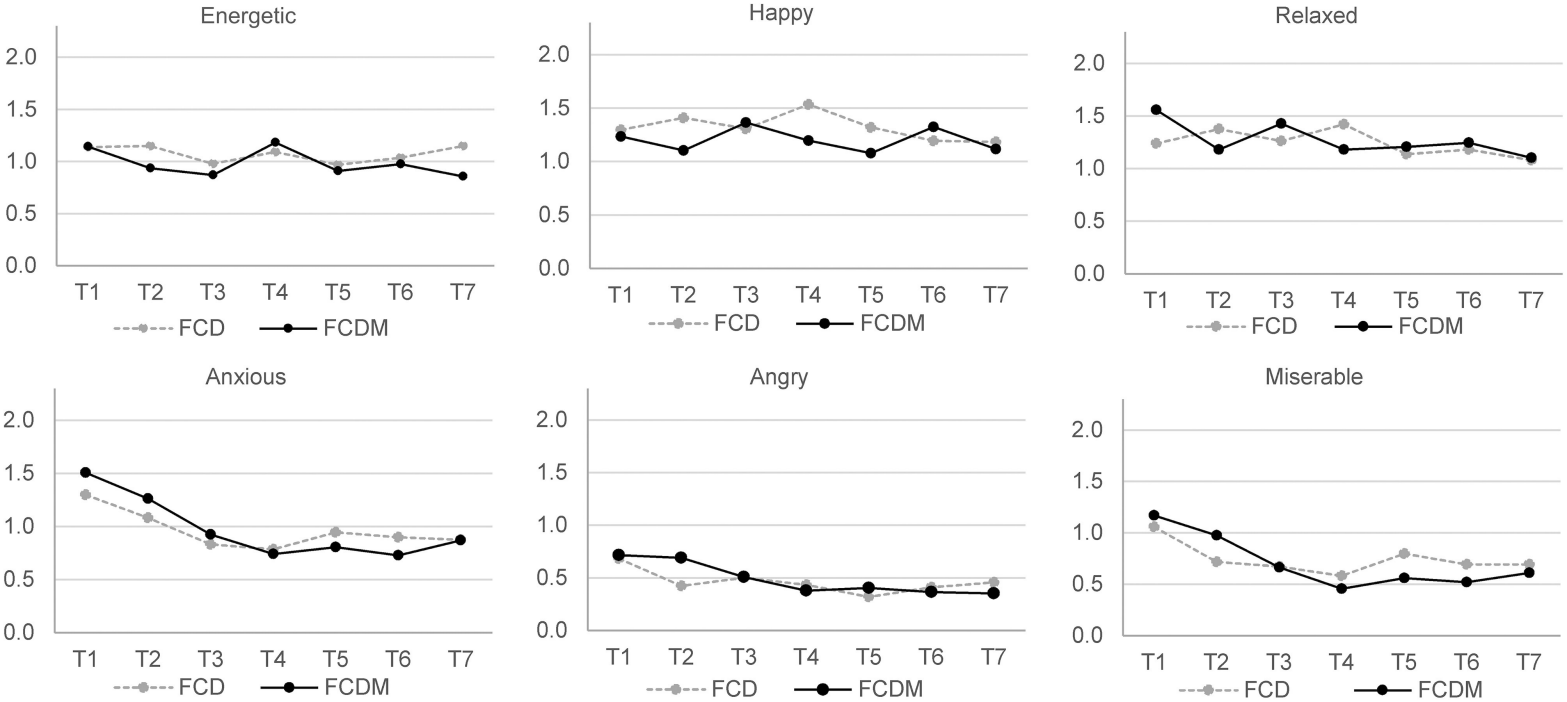
Intensity of emotional states associated with strongest food cravings across a 7-day intervention (FCD, *n* = 88; FCDM, *n* = 77).

In regards to the emotions experienced at the time of the highest food cravings, a repeated measures MANOVA yielded significant main effects of Time, Wilk's λ = 0.374, *F*_(54, 110)_ = 3.404, *P* < 0.001, $\eta_{\text{p}}^{2}$ = 0.626. No significant differences were observed for Group main effect, Wilk's λ = 0.978, *F*_(9, 155)_ = 3.384, *P* = 0.941, $\eta_{\text{p}}^{2}$ = 0.022, and Time by Group interaction, Wilk's λ = 0.622, *F*_(54, 110)_ = 1.240, *P* = 0.171, $\eta_{\text{p}}^{2}$ = 0.378. Reported emotional states intensities for each group are presented in Figure 3. *Post hoc* analysis on the main effects of Time indicated that individuals reported significantly lower intensities for all unpleasant states (i.e., angry, anxious, miserable, tired, bored, frustrated) across time. Differences in the intensities of pleasant states reported across the intervention were not significant (see Supplementary Table S3 for significant pairwise comparisons).

### Perceived Changes in Eating and Wellbeing, Physical and Mental Health

Descriptive statistics for perceived changes in eating, wellbeing, physical health, and emotional health reported pre-intervention (T1) and post-intervention (T7) are presented in Table 2. MANOVA yielded significant main effects of the Intervention (pre-, post), Wilk's λ = 0.603, *F*_(4, 155)_ = 25.530, *P*< 0.001, $\eta_{\text{p}}^{2}$ = 0.397. The main effects of Group, Wilk's λ = 0.994, *F*_(4, 155)_ = 0.226, *P* = 0.923, $\eta_{\text{p}}^{2}$ = 0.006 or the Intervention by Group interaction, Wilk's λ = 0.972, *F*_(4, 155)_ = 1.129, *P* = 0.345, $\eta_{\text{p}}^{2}$ = 0.028 were not significant. *Post hoc* analysis indicated that participants reported eating significantly less, *F*_(1, 158)_ = 76.428, *P* < 0.001, $\eta_{\text{p}}^{2}$ = 0.326), and perceived enhanced wellbeing, *F*_(1, 158)_ = 57.239, *P* < 0.001, $\eta_{\text{p}}^{2}$ = 0.266 at the end of the study.

**Table 2:** Reported changes in eating, wellbeing, physical and emotional health at T1 and T7 for FCD and FCDM group participants.

		**T1**	**T7**
**Variable**	**Group**	***M (SD)***	***M (SD)***
Eating (-5, +5)	FCD	1.06 (2.1)	−0.2 (1.2)
	FCDM	1.49 (1.7)	−0.4 (1.4)
Wellbeing (−5, +5)	FCD	−0.64 (2.2)	0.7 (1.1)
	FCDM	−0.71 (2.0)	0.7 (1.2)
Physical health (1–6)	FCD	3.80 (1.1)	3.7 (0.9)
	FCDM	3.79 (1.1)	3.9 (1.1)
Emotional health (1–5)	FCD	2.47 (1.2)	2.5 (1.1)
	FCDM	2.55 (1.1)	2.5 (1.0)

### Perceived Easiness and Effectiveness

Regarding the food craving diary, perceptions of easiness in completing it were positive for participants in both FCD (*M* = 1.87, *SD* = 1.35), and FCDM (*M* = 1.62, *SD* = 1.61) groups. Participants' perceptions of its effectiveness in helping them manage food cravings ranged from −3 to +3 (*M* = −0.22, *SD* = 1.58) for FCD group participants, and from −3 to +3 (*M* = 0.18, *SD* = 1.34) for FCDM group participants. MANOVAs did not yield significant main effects of Group in easiness and effectiveness of the food craving diary, Wilk's λ = 0.969, *F*_(2, 158)_ = 2.555, *P* = 0.081, $\eta_{\text{p}}^{2}$ = 0.505. Regarding mindful eating practice, FCDM group participants' perceptions of easiness in carrying it out ranged from −3 to +3 (*M* = 0.89, *SD* = 1.62). FCDM participants felt diary completion to be easier compared to the practice of mindful eating, *t*(73) = 3.595, *P* < 0.001. Moreover, mindful eating practice was perceived as more effective than diary completion, *t*(73) = −2.945, *P* = 0.004.

### Participant Feedback

Participants, who provided comments at the end of the study, indicated that they perceived completion of the food craving diary to be an easy task, as the following comment exemplifies:

*The diary was very easy to complete- bit wary as it is an area that I have work to do however I am also conscious that it could produce very difficult emotions and which is why I have avoided it. This was a gentle and non-invasive way to start looking at these issues- bringing attention to them without judgement or action plan etc*.

They also noted how the food craving diary helped raise their awareness of emotionally elicited eating prompting them to initiate strategies for healthier eating, illustrated as follows: “*It allowed me to identify negative eating behaviors. I now have fruit at hand always so I eat that instead of finding chocolate if I'm hungry or stressed or bored. So feel like it actually helped”, “During lockdown we have managed the stress with food and the study has been good to help control the cravings”*, and:

*I had already decided to log my food intake and this study made me think more about when I want to snack and why. Boredom and relaxation really impact my food intake. Keeping busy helps me manage my food consumption. Thank you for helping me with this insight*.

Some participants reported the mindful eating practice to be “*very easy to follow and understand”* and “*not very time-consuming*”. However, feedback also described a need to feel confident in using the strategy: “*Becoming aware of the emotions experienced every time we have a craving and how to manage the feelings in those situations is easier than actually doing it”* and:

*Personally, I think this study has helped me to see the link between emotions and the “need” of snacking. To become aware of this link is a very good first step, however, I have found the mindfulness input very low, especially if someone is not familiar with this concept*.

Mindful eating practice was perceived by some to be a strategy they could, and indeed would like to continue to implement after the completion of the study “*…it helped me to eat more consciously, with more attention. I will continue practicing mindful eating.”* Indeed, several participants expressed a desire to continue using the interventions provided:

*Interesting study that drew attention to the influence of one's own emotions. It felt like the week was just getting started - I would like to continue filling out the diary for a longer period of time, or to take part in a longer study*.

*This was brilliant!! Having ‘let myself go' during the lockdown it was just what I needed to kick start healthy eating again. I have lost a total of 5lb this week, and have downloaded an app to track my eating habits to continue progress*.

## Discussion

This was a randomized pilot study with two conditions (i.e., completion of a food craving diary and completion of food craving diary and mindful eating practice) that examined the feasibility of implementing a remote intervention aimed to manage food cravings and associated emotional experiences during the COVID-19 pandemic. This 7 days remote study was implemented fully online with no contact between participants and researchers. Although limited by the lack of a control group, the study results indicated significant changes in some of the targeted variables. Contrary to our hypothesis, no significant differences were observed due to following mindful eating practice guidelines in addition to completion of a food craving diary.

Descriptive data indicate that tired and bored were the two most intensely experienced unpleasant emotions for participants in both groups at T1. In seeking to explain this finding, it is widely reported that containment measures applied during the pandemic reduced the number of accessible daily activities and enforced changed routines. For example, congregating or mixing with others was no longer permissible, clubs and organized face-to-face activities ceased. As a result, research suggests that time was perceived as slowing down for many when containment measures were in effect, and this conscious experience of time increased feelings of boredom and sadness (Droit-Volet et al., 2020).

Whilst there were no significant between group differences in emotions, there was a significant reduction in the intensity of all unpleasant emotions experienced associated with cravings from T1 to T7 (Figure 3). This suggests that the act of completing a daily diary to identify emotional states associated with food cravings may have increased participants' awareness of felt emotions, thereby triggering emotion regulation. When experiencing unpleasant emotions, individuals typically engage in hedonic emotion regulation, characterized by trying to increase the intensity of pleasant emotions and reduce the intensity of unpleasant emotions, unless they believe unpleasant emotions are useful, thereby accommodating utilitarian considerations in emotion regulation (Tamir et al., 2007). The present study suggests that regulation efforts among participants were driven by hedonic motives toward reducing the intensity of unpleasant emotions.

Whilst there was no overall difference between groups in terms of food craving reduction, it is informative to examine patterns in the data, as the reported craving outcomes follow a different pattern over time for each group. Observing these patterns may be helpful in hypothesizing about the function of a combined diary and mindful eating intervention in bringing about behavioral change over time. Observing FCD group participants who received the food craving diary only, there is a significant reduction in the frequency of cravings, giving in to cravings and difficulty in resisting cravings at T2. By contrast, FCDM group participants who received the food craving diary plus mindful eating intervention evidenced a steadier reduction over time. Participants reported greater ease in using the craving diary as compared to the mindful eating intervention. Most participants providing feedback on their experiences with diary completion noted how it increased their awareness of emotions that elicit cravings. Many participants then detailed strategies they used to help manage this. FCD group participants, having no guidance on strategies to manage emotionally elicited cravings, likely self-selected strategies they felt confident in utilizing. As such, they did not have the need to learn a researcher prescribed and potentially novel strategy.

FCDM group participants were asked to use mindful eating as a strategy to help manage emotional eating. Whilst we did not evaluate previous experience with mindful eating, we can speculate that for many participants this presented a novel intervention. This novelty might explain the difficulties in complying with this practice, which was reported by some participants and may also account for differences at T2. T2 data suggests that participants in the craving diary only condition showed greater reductions in cravings, acting on cravings, and difficulties in resisting cravings. They also experienced greater reductions in unpleasant emotion. People often struggle to adhere to psychological skills training programmes (Shambrook and Bull, 1999); notably where they find the intervention difficult to follow. This alludes to the possibility that the intervention process is effortful and so might not lead to immediate benefits. Furthermore, where an intervention is perceived as more difficult to follow, an individual may perceive effort invested as producing insufficient benefits. This highlights the importance of setting expectations for intervention use, and setting realistic outcome expectancies, in particular during the early stages of the intervention. It also suggests that for mindful eating, guided practice by an experienced trainer may be necessary to initiate and facilitate practice, rather than the exclusive use of remote online means of intervention delivery and support. Irrespective of intervention condition, unpleasant emotions were stronger triggers for cravings at the beginning but decreased over the week. Happiness as a trigger for food cravings remained the same.

A final point of discussion relates to participant attrition. A high dropout rate (76%) was observed from the initial assessment (*n* = 2075) to the first follow-up food craving diary (*n* = 488). The rate of attrition decreased across the latter stages of the intervention (see Figure 1). Analysis also indicate that recruitment of male participants was lower, and attrition higher. This is in line with previous research undertaken on emotional eating where significant differences in the recruitment and retention of male and female participants have been reported. A systematic review of 14 studies on mindfulness-based interventions for emotional eating (Katterman et al., 2014) included 5 studies who recruited exclusively female samples, and of the 9 remaining, males only represented between 10–37% of the total sample. A similar pattern is observed in studies of related concepts such as weight management. For example, a systematic review of 244 randomized controlled trials of weight loss programmes (*n* = 95,207) found that only 27% of participants were men (Pagoto et al., 2012). Research indicates that men perceive weight loss services to be feminized spaces, in which they feel self-conscious and out of place (Elliott et al., 2020). When the contexts of weight and emotion combine, as with emotional eating, then perceptions of this as a “feminized space” may intensify. Emotion is a term that has long been associated with the personal and the feminine (Åhäll, 2018). Thus, interventions based around these concepts may be met with resistance from men due to mismatch with ideologies of masculinity (Isacco, 2015).

Previous research has evidenced high rates of study attrition when delivering remote online interventions (Christensen et al., 2009). Existing explanations for high attrition with remote interventions include a need to provide brief support that supplements remote self-help (Richards and Richardson, 2012) and the ease of access to online interventions which invite browsing and curiosity (Mason et al., 2018). There is qualitative evidence to suggest that some participants found the mindful eating intervention to be the more challenging of the two interventions and would have appreciated additional guidance for its use. As such, for these individuals, perceived difficulty of use may have contributed to a decision to cease participation. However, we propose two further plausible explanations. The first explanation is informed by data from the present study. Figures 2, 3 respectively show the greatest decrease in food cravings (experiencing, giving in to, and difficulty resisting), and unpleasant emotions (anxious, angry, miserable, tired, and frustrated) from T1 to T2. At this point, participants had experienced the craving diary or craving diary plus mindful eating intervention. Qualitative data indicate that most participants found the interventions easy to follow, and experienced early benefit, in particular for the food craving diary. It is plausible that facilitated by the remote nature of the research, some participants felt under no obligation to continue with the research once they fulfilled their goals for participation. In other words, where participants perceived they had adequately (by their own self-referenced standards) recognized and regulated unpleasant emotion and associated unwanted food cravings, some may have perceived no further incentive for ongoing participation. The second explanation is informed by the context of the present study, in particular, experiences of “zoom fatigue” commonly reported during COVID-19. It is likely that for some participants, a requirement for daily online diary and survey completion was de-incentivising due to high levels of online fatigue, with priorities for screen time allocated to work and social activities. Likely a combination of all the aforementioned explanations contributed to participant attrition, and consideration should be given to each in designing remote interventions and recruiting participants. For example, by incorporating measures associated with adherence (e.g., motivation for change), establishing means to differentiate curious browsers from those with true interest in the study, and keeping screen time brief and informative. It is worth noting that in the present study participant attrition from day 3 assessment onwards reduced dramatically (Figure 1). This suggests that following initial curiosity, where participants were able to establish congruence with their own goals for involvement this was sufficient to ensure ongoing adherence. Indeed, some participants reported that they intended to continue with the interventions provided after the cessation of participatory requirements.

### Limitations and Future Research Directions

One aim of this study was to provide participants with brief and easy strategies to regulate their emotions and associated food cravings, which seemed to be appropriate considering the reported changes in eating behaviors during the COVID-19 pandemic (Ruiz et al., 2021). There are several advantages related to the remote format of delivery, which allowed access to geographically dispersed participants with different backgrounds and ages. In this study, the use of technology also facilitated sending daily reminders to participants. However, one of the limitations of daily retrospective assessments is recall bias, where participants may recall more intense experiences rather than those that endure for longer time (Gunthert and Wenze, 2012). Future interventions could use ecological momentary assessment (EMA) (Shiffman, 2009) in which participants are sent repeated cues for self-reporting across the day. This is one of the most advanced methods of recording self-monitoring, and has been advocated for in a systematic review on emotional eating in normal and overweight individuals (Devonport et al., 2019).

The lack of significant differences in food cravings between the two groups may be explained by the individuals' prior experiences of and current engagement in mindfulness practice or with mindful eating practice outside of the study, which was not captured, and thus equivalence across groups cannot be assumed. We recommend that future research captures prior mindfulness training or practice and experience with mindful eating to account for this potential confounding factor.

A further limitation of the present study is that we did not ask participants to report on their perceived need to manage emotionally elicited cravings, or their compliance with the mindful eating guidelines. The extent to which people engaged with the guidelines provided would likely vary and may largely depend on their previous experience. Whilst we did not complete a manipulation check, we can draw on participant feedback which points to a difference in experience between the two groups. Participants found mindful eating a harder intervention to follow. Whilst we provided written instructions for the use of mindful eating, it may be that a more immersive experience (e.g., video or audio recordings) with opportunities for question and answers, would have increased the ease of intervention use.

The use of a web-based intervention meant reliance on individuals coming across recruitment information via social media, then completing the baseline survey on a voluntary basis. With COVID-19 related restrictions, this was the only viable form of administration. However, there are groups with known issues regarding digital literacy and accessibility to web-based research including some older adults, people with long-term health conditions or disabilities, and those without internet access. These groups are therefore likely to be under-represented in the present study. Furthermore, the present study recruited few male participants, which mirrors the pattern in weight management studies generally, where typically less than 30% of participants are male (Tsai and Wadden, 2005). Caution must therefore be exercised in generalizing findings of the present study to these groups, and consideration should be given as to how future research could recruit male participants, and therefore better account for the male experience. Male-only support groups seem to be effective (Young et al., 2012), especially those delivered virtually (Azar et al., 2015).

The results provide initial support for the use of remote interventions to help manage food cravings and associated emotional experiences. Participants reported that the food craving diary was easy to complete. Knowing that this is an accessible, easy, and effective intervention for the reduction of food cravings and related eating behavior could have wide reaching applications in the provision of remote healthcare. The need for healthcare to move to a blended form and better utilize technological resources is being increasingly recognized (Nicholls et al., 2016). The delivery of effective remote interventions such as those used in the present study has potential to expedite the provision of healthcare interventions. This is especially critical at a time when healthcare services worldwide are under pressure due to pandemic related reductions in service provisions (Brown et al., 2021).

## Conclusions

Significantly less eating and better wellbeing were reported after both intervention conditions. The completion of a 7-day food craving diary was effective in reducing food cravings (i.e., frequency of craving experienced, giving into craving, and difficulty resisting), as well as the intensities of unpleasant emotions experienced at the time of the highest food cravings. Following mindful eating guidelines alongside a food diary for the same time period was no more effective than completion of the diary alone. Our findings highlight the need to consider ways to increase participant involvement and retention.

## Data Availability Statement

The raw data supporting the conclusions of this article will be made available by the authors, without undue reservation.

## Ethics Statement

The studies involving human participants were reviewed and approved by University of Wolverhampton Ethics Committee (Unique code: 01/20/AF1/UOW). The participants provided their written informed consent to participate in this study.

## Author Contributions

TD, C-HC-W, and MR: conceptualization and methodology. C-HC-W: data management. C-HC-W and MR: data curation. CR and MR: formal analysis. TD, C-HC-W, WN, and MR: writing – original draft. TD, C-HC-W, WN, JC, CR, JF-M, YC, and MR: writing – review and editing. All authors contributed to the article and approved the submitted version.

## Conflict of Interest

The authors declare that the research was conducted in the absence of any commercial or financial relationships that could be construed as a potential conflict of interest.

## Publisher's Note

All claims expressed in this article are solely those of the authors and do not necessarily represent those of their affiliated organizations, or those of the publisher, the editors and the reviewers. Any product that may be evaluated in this article, or claim that may be made by its manufacturer, is not guaranteed or endorsed by the publisher.
