# Supplementary material for: Brief Remote Intervention to Manage Food Cravings and Emotions During the COVID-19 Pandemic: A Pilot Study
Source: Front Psychol. 2022 Jun 30;13:903096. doi: 10.3389/fpsyg.2022.903096 (PMC9280415; doi:10.3389/fpsyg.2022.903096)
Supplement: Supplementary file 1 [file Data_Sheet_1.PDF]

Supplementary Table S1. Pearson product-moment correlation coefficients for the studies variables at T1 (N =165)

Variable	1	2	3	4	5	6	7	8	9	10	11	12	13	14	15
1. Eating															
2. Wellbeing	-.15														
3. Physical health	-.20	.45													
4. Impact on emotional health	.20	-.41	-.47												
5. Craving experience	.26	-.15	-.09	.21											
6. Give in to cravings	.35	-.11	-.13	.26	.55										
7. Difficulty resisting cravings	.33	-.16	-.12	.13	.51	.67									
8. Energetic	.14	.02	-.17	.09	.15	.04	.00								
9. Relaxed	.12	.05	-.16	.08	.03	.15	.08	.54							
1. Happy	.12	.05	-.15	.10	.08	.08	.07	.71	.65						
11. Anxious	.21	-.18	-.11	.28	.40	.28	.24	-.10	-.16	-.03					
12. Angry	.18	-.04	-.01	.10	.21	.12	.15	.09	-.05	.16	.54				
13. Miserable	.14	-.18	-.13	.28	.32	.30	.31	-.09	-.08	-.01	.57	.52			
14. Tired	.09	-.07	-.06	.12	.30	.28	.30	-.03	-.02	-.05	.40	.21	.46		
15. Bored	.34	-.06	-.11	.11	.27	.32	.28	-.06	.15	.05	.16	.08	.26	.19	
16. Frustrated	.21	-.18	-.15	.35	.38	.27	.27	-.05	-.12	.05	.63	.55	.66	.43	.18

Note. .0–.19 = no correlation, .20–.39 = low correlation, .40–.59 = moderate correlation, .60–.79 = moderately high correlation, and > .80 = high correlation.

Supplementary Table S2. P-values of pairwise comparisons for food cravings (n = 165).

		T1	T2	T3	T4	T5	T6
Food cravings experience	T1						
	T2	.001					
	T3	.001	.911				
	T4	.001	.006	.673			
	T5	.001	.029	.736	1.000		
	T6	.001	.264	.994	1.000	1.000	
	T7	.001	.092	.962	1.000	1.000	1.000
Frequency giving into cravings	T1						
	T2	.001					
	T3	.001	1.000				
	T4	.001	.662	1.000			
	T5	.001	.270	.883	1.000		
	T6	.001	.904	1.000	1.000	1.000	
	T7	.001	.177	.867	1.000	1.000	1.000
Difficulty resisting cravings	T1						
	T2	.001					
	T3	.003	1.000				
	T4	.001	.946	.993			
	T5	.001	1.000	1.000	1.000		
	T6	.003	1.000	1.000	.991	1.000	
	T7	.001	1.000	1.000	1.000	1.000	1.000

Note. Šidák adjustment for pairwise comparisons was used.

Supplementary Table S3. P-values of pairwise comparisons for unpleasant emotional states associated with strongest cravings (N = 165)

		T1	T2	T3	T4	T5	T6
Angry	T1						
	T2	.651					
	T3	.101	1.000				
	T4	.002	.509	.946			
	T5	.001	.079	.115	1.000		
	T6	.002	.265	.892	1.000	1.000	
	T7	.002	.691	.989	1.000	.999	1.000
Anxious	T1						
	T2	.802					
	T3	.001	.041				
	T4	.001	.001	.977			
	T5	.001	.075	1.000	.987		
	T6	.001	.029	1.000	1.000	1.000	
	T7	.001	.076	1.000	.996	1.000	1.000
Miserable	T1						
	T2	.097					
	T3	.001	.626				
	T4	.001	.005	.302			
	T5	.001	.872	1.000	.536		
	T6	.001	.578	1.000	.971	1.000	
	T7	.001	.805	1.000	.712	1.000	1.000
Tired	T1						
	T2	.982					
	T3	.025	.545				
	T4	.034	.469	1.000			
	T5	.008	.499	1.000	1.000		
	T6	.020	.637	1.000	1.000	1.000	
	T7	.002	.260	1.000	1.000	1.000	1.000
Bored	T1						
	T2	.001					
	T3	.001	.962				
	T4	.001	.304	1.000			
	T5	.001	.010	.657	.955		
	T6	.001	.004	.337	.742	1.000	
	T7	.001	.001	.010	.108	.941	1.000
Frustrated	T1						
	T2	.488					
	T3	.033	.992				
	T4	.001	.002	.022			
	T5	.001	.007	.107	1.000		
	T6	.001	.001	.029	1.000	1.000	
	T7	.001	.048	.532	1.000	1.000	.955

Note. Šidák adjustment for pairwise comparisons was used. Pairwise comparisons for pleasant emotional states (i.e., energetic, relaxed, happy) were not significant.
